# Oowana: A New Toilet Soap

**Published:** 1902-08-23

**Authors:** 


### Editor's Letter-Box.

[Our Correspondents are reminded that prolixity is a great bar to publication, and that brevity of style and conciseness of statement greatly facilitate early insertion.]

### OOWANA: A NEW TOILET SOAP.

Commenting on a notice of Oowana which appeared in The Hospital on August 9th, the proprietors write: "The soap *is entirely free* from animal matter of any description. We even avoid perfumes which come from animal life. The oils are purely vegetable."

